# Influential nodes identification in complex networks: a comprehensive literature review

**DOI:** 10.1186/s43088-023-00357-w

**Published:** 2023-02-14

**Authors:** Khaoula Ait Rai, Mustapha Machkour, Jilali Antari

**Affiliations:** 1 Computer System and Vision Laboratory, Faculty of Sciences Agadir BP8106, Ibn Zohr University, Agadir, Morocco; 2 Laboratory of Computer Systems Engineering, Mathematics and Applications, Polydisciplinary Faculty of Taroudant, Ibn Zohr University, B.P. 8106, Agadir, Morocco

**Keywords:** Complex networks, Network measurements, Network structure, Social influence influential nodes, Network models

## Abstract

Researchers have paid a lot of attention to complex networks in recent decades. Due to their rapid evolution, they turn into a major scientific and innovative field. Several studies on complex networks are carried out, and other subjects are evolving every day such as the challenge of detecting influential nodes. In this study, we provide a brief overview of complex networks, as well as several concepts key related to measurements, the structure of complex network and social influence, an important state of the art on complex networks including basic metrics on complex networks, the evolution of their topology over the years as well as the dynamic of networks. A detailed literature about influential finding approaches is also provided to indicate their strength and shortcomings. We aim that our contribution of literature can be an interesting base of information for beginners’ scientists in this field. At the end of this paper, some conclusions are drawn and some future perspectives are mentioned to be studied as new directions in the future. More detailed references are provided to go further and deep in this area.

## Background

The study of complex networks has been the subject of great attention from the scientific community and has proved useful in many fields such as physics, biology, telecommunications, computer science, sociology and epidemiology. Complex networks (CN) become a major scientific research field. In our daily life, there are several examples of complex networks. For instance, the world wide web is a real network composed of web pages connected by hypertext links; internet is a network of computers and routers attached by optical fibers; metabolic networks is a network of interaction between metabolites; neural networks represent simple neurons in brain linked to form a complex system. Such CN and others can be modeled as graphs composed of nodes that interact with each others, and the interaction between nodes is presented by links or edges. Graph theory is a powerful tool that has been employed in a variety of complex network studies [1, 2]. The modeling of these systems allowed us to explore them, to understand their mathematical description, to understand their various behavior and to predict it. The modeling consists of creating coherent models that reflect the properties of real networks as much as possible. In real networks, while local interactions are well known such as the communication between routers and the protein–protein interaction, the overall result of all the interactions is still poorly understood (emergence property). For a better understanding of the characteristics of networks, we will need a formalism that encompasses the structure of the network (static approach) and its function (dynamic approach) [3]. The analysis of complex networks relies on knowing some fundamental concept such as network measurements, network structure, and social influence.

Models and real networks can be compared using network measurements. These measurements can express the most suitable topological features and can be an efficient source for networks investigation. Clustering coefficient, average path length, and degree distribution are some statistical measurements that can define the structure and the behavior of networks. An overview about these measurements is provided in Sect. 2. The structure of a network means the way each node is arranged. It is the underlying layer of network’s dynamics [4, 5]. Analyzing the dynamic of networks allows us to find out different behaviors of networks either in static or variable state.

With the study of network structure, the identification of influential nodes and the detection of community are an important issues that have recently been dealt by the scientific community. The detection of community is addressed in a range of methods. Each method has its own characteristics. The second issue is determining which nodes in networks are important; different approaches are proposed to fix this challenge. These approaches are divided into four categories: structured approaches (local, semi local, global and hybrid methods), Eigen vector-based approaches which rely on the quantity of neighbors and their influences, multi-criteria decision making (MCDM)-based approaches and machine learning-based approaches. Each method has its limitations. There are methods that consider local network information or methods that consider global information or methods that rely on feature engineering and the selection of this features. We give later a detailed comparison summary table of some used approaches to extract similarities and differences between them.

The main contributions of this paper are the presentation of a relevant state-of-the-art review on complex networks, and all concepts related to them like measurements, structure, social influence, and especially the influential node approach. A comprehensive review and categorization of different approaches used in influential node findings are presented to highlight their main advantages and weaknesses. Hoping that this paper will help scientists with the analysis in this field.

The rest of this paper is organized as follows: Sect. 2 provides the main text of the manuscript, a quick review of our subject's fundamental concepts is provided in Sect. 2.1. An interesting literature review about complex networks is highlighted in Sect. 2.2. The third sub-section discusses methods for detecting influential nodes. Section 4 is a summary of the classification of several papers. In Sect. 3, we draw conclusions and some perspectives.

## Main text

### Fundamental concepts

In this section, we present some basic concepts and definitions that will be used in this article.

#### Complex network

In the context of network theory, CN is a network of interactions between entities whose overall behavior is not deductible from the individual behaviors of the said entities, hence the emergence of new properties. It refers to all entities that are linked to each other in some way. In other word, CN is a graph (network) with nontrivial topological features, features that do not occur in simple networks such as random networks, but often occur in networks representing real systems. The study of complex networks is a young and active field of scientific research largely inspired by the empirical findings of real-world networks such as:

- *Social networks* A social network, such as Facebook or Twitter, is a collection of social actors, such as persons or groups, connected by social interactions. It is a set of vertices and edges that describes a dynamic community.
- *Biological networks* for example, metabolic networks with proteins as nodes and chemical interactions as links.
- *Infrastructure networks* for example, transport networks whose nodes are airports and the links are air links as well as electricity networks (cables between places of production and consumption).

**Fig. 1:**
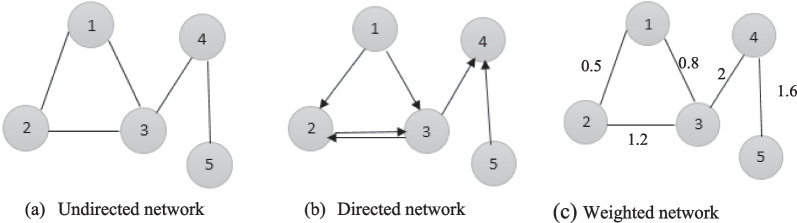
Examples of networks

Most social, biological, and technological networks exhibit substantial non-trivial topological features, with connection patterns between their elements that are neither purely regular nor purely random [6]. These characteristics include a heavy tail in the degree distribution, a high clustering coefficient, assortativity or dissortativity between vertices, community structure, and hierarchical structure. In the case of directed networks, these characteristics also include reciprocity, triad importance profile, and other characteristics [7]. In contrast, many mathematical models of networks that have been studied in the past, such as networks and random graphs, do not exhibit these characteristics. The most of complex structures can be realized by networks with an average number of interactions. It is often possible to predict the functionality or understand the behavior of a complex system if we can verify certain "good properties" by analyzing the underlying network [8]. For example, if we detect clusters of vertices with the same topological characteristics of the network, we can obtain information about the particular roles played by each vertex (e.g., hubs, outliers) or how whole clusters describe or affect the general behavior of the CN [9]. The use of graph theory to model networks as graphs makes it easier to examine and understand their structure. Graphs are used to model this, with nodes representing entities and links representing relationships. A graph G is a couple $\left(V,E\right)$ where: $V={v}_{1},{v}_{2},\dots {,v}_{n}$ such as $n=\left|v\right|$ is a set of vertices or nodes. $E={e}_{1},{e}_{2},\dots .{,e}_{m}$ such as $m=\left|e\right|$ is a set of edges or links. If each edge $E$ is an unordered pair of nodes, the edge is undirected and the network is an undirected network. Otherwise, if each edge is an ordered pair of nodes, the edge is directed from node to other and the network is a directed or oriented network. In this case, an ordered pair of nodes $(u,v)$ is a directed edge from node $u$ to node $v$. If each edge has an associated numeric value called a weight, the edge is weighted and the network is a weighted network [10]. Figure 1 shows three examples of networks including undirected, directed and weighted network (undirected).

**Fig. 2:**
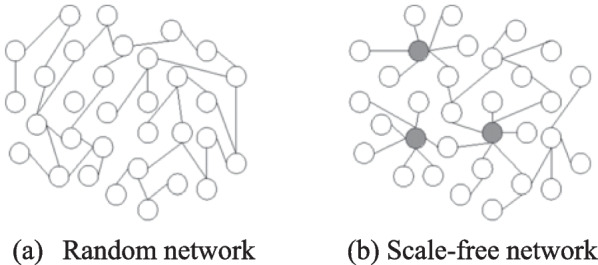
The random network and scale free network **a** the random network is virtually homogeneous and follows the Poisson distribution. Nearly all nodes have the same number of links. **b** Scale-free network: An inhomogeneous network that exhibits power-law behavior. The majority of nodes have one or two links, but a few densely connected nodes, or "hubs," have many links.in the scale-free network, the largest hubs are highlighted with dark circles, nodes are presented with white circles [18]

Two well-known and much-studied classes of complex networks are scale-free networks [11, 12] and small-world networks [13, 14], whose discovery and definition are canonical case studies in the field. Both are characterized by specific structural features: power-law degree distributions for the first class [15], short path lengths and high clustering for the second class. Examples of these classes are presented in Fig. 2. The random network is virtually homogeneous and follows the Poisson distribution. Nearly all nodes have the same number of links. Road network is an example of this class. scale-free network: An inhomogeneous network that exhibits power-law behavior. The majority of nodes have one or two links, but a few densely connected nodes, or "hubs," have many links. Airline networks is an example of this class. However, as the study of complex networks has continued to grow in importance and popularity, many other aspects of network structures have also attracted attention [16, 17]. Section 3 presents these classes with their characteristics.

Recently, the study of complex networks has been extended to networks of networks. If these networks are interdependent, they become significantly more vulnerable than single networks to random failures and targeted attacks and exhibit cascading failures and first-order percolation transitions [19]. In addition, the collective behavior of a network in the presence of node failure and recovery has been studied. It has been found that such a network can have spontaneous failures and spontaneous recoveries [20].

The field continues to grow at a rapid pace and has brought together researchers from many fields, including mathematics, physics, biology, computer science, sociology, epidemiology, and others. Ideas and tools from network science and engineering were applied to the analysis of metabolic and genetic regulatory networks; the study of the stability and robustness of ecosystems; clinical science; modeling and design of scalable communication networks such as generation and visualization of complex wireless networks; the development of vaccination strategies for disease control; and a wide range of other practical issues [21]. In addition, network theory has recently proven useful in identifying bottlenecks in urban traffic. Network science is the subject of many conferences in a variety of different fields [22].

#### Network measurements

In the field of complex network, measurements can demonstrate the most relevant topological features, especially after the representation of the network structure, the analysis of the topological characteristics of the obtained representation carried out in the form of a set of informative measurements. During the modeling process, some respective measurements are used for comparing models with real networks. That is why it is an essential resource in many network investigation [23].

Thereafter, some measurements that can be used to measure significant properties of complex systems. We consider then a graph $G$ with $G\left( {V,E} \right)$. $V$ is a set of nodes, and $E$ is a set of edges.

*Density *the density d of a graph $G$ is the proportion of links existing in $G$ compared to the total number of possible links: $\left( G \right) = 2m/n\left( {n - 1} \right)$. If $m$ is of the order of $n$, the graph is said to be sparse (as opposed to dense graphs). Indeed, this measure is sensitive to the number of vertices, so the density equal to $0$ corresponds to the graph where all the vertices are isolated, and equal to $1$ in the case of a complete graph. In a graph resulting from empirical observations, the more the number of vertices increases, the more the density tends to decrease.

*Shortest path* it is the length of the shortest path connecting two nodes in the network. One of the algorithms for calculating the distance between two nodes in a graph is: Dijkstra's algorithm [24]. The average distance between two pairs of nodes makes it possible to evaluate the transmission time required between two “any” individuals.

*Diameter* the diameter of a network is formally the longest of the shortest paths between two entities, or nodes, of the network, via its connections. It allows for example to know the maximum time to transmit the disease.

*Degree* the degree $d\left( i \right)$ of a node is the number of edges incident to node $i$, in other words, the number of neighboring nodes of $i$.

*Degree distribution* perhaps calculated as follows [25]:

1 $$P\left( k \right) = \frac{{\left| {\delta \left( v \right)} \right|}}{N}$$

$\delta \left( v \right)$ denotes the number of vertices of the network $G$ having degree $k$ and $N$: denotes the size of $G$ (number of nodes). The above equation represents the proportion of vertices of $G$ having degree $k$. The degree $k_{i}$ of node $i$ is the number of links connected to node $i$. The distribution of degrees allows the understanding of the distribution of connectivity and the structure of the network.

*Clustering coefficient* is the probability that two neighbors of a node are also neighbors to each other. It can be interpreted as the probability that two nearest neighbors of $i$ are connected to each other.

*The average clustering coefficient* of the graph $G$ is the average of the clustering coefficient of all its vertices (nodes). In the literature, there are two definitions of the clustering coefficient: global clustering coefficient (also called transitive) and local clustering coefficient on average [26]:

The global clustering coefficient is defined as:

2 $$C = \frac{{3*{\text{number}}\;{\text{of}}\;{\text{triangles}}}}{{{\text{number}}\;{\text{of}}\;{\text{connected}}\;{\text{triplets}}}}$$

With:

A triangle is a complete subgraph with three nodes;

A connected triple is a set of three vertices with at least two links between them.

*Closeness centrality* indicates if a node is located close to the all other nodes of the graph and if it can quickly interact with them. It is written formally [27]:

3 $$C_{c} \left( v \right) = \frac{1}{{\mathop \sum \nolimits_{{u \in V \in \backslash \left\{ v \right\}}} d_{G} \left( {u,v} \right)}}$$

With $d_{G} \left( {u,v} \right)$ is the distance between nodes $u\;{\text{ and}} \;v.$

*Betweenness centrality* is one of the most important concepts. It measures the usefulness of the node in the transmission of information within the network. The node plays a central role if many shortest paths between two nodes have to go through this node [27]. Formally, we express it as:

4 $$C_{B} \left( v \right) = \mathop \sum \limits_{{\begin{array}{*{20}c} {i,j} \\ {i \ne j \ne v} \\ \end{array} }} \frac{{\sigma_{ij} \left( v \right)}}{{\sigma_{ij} }}$$

with $\sigma_{ij} \left( v \right)$ the number of paths between $i$ and $j$ that go cross $v.$

*Vulnerability* A node’s vulnerability is defined as the decrease in performance that occurs when the node and all of its edges are removed from the network.

5 $$V_{i} = \frac{{E - E_{i} }}{E}$$

where $E$ is the original network's global efficiency and $E_{i}$ is the global efficiency after omitting node $i$ and all its edges.

6 $$E = \frac{1}{{N\left( {N - 1} \right)}}\mathop \sum \limits_{i \ne j} \frac{1}{{d_{ij} }}$$

#### Complex network structure

The way in which the nodes are arranged is another aspect in the study of the complex networks or the study of their structure. The structure refers to the real-world network modeling research that has been done. Several models, however, appear to explain how small world networks and scale-free features emerge in the real world: Watts and Strogatz proposed a model [26] to explain how the two characteristics of small world networks, a high clustering coefficient and a low average path length, expound in networks. Barabàsi and Albert offered a model [28] to show how networks with power-law degree distribution emerge in networks.

Usually, models of networks can help us to understand the meaning of these properties, we can classify these models in two categories:

- *Evolving models* explains the evolution of the complex network as a function of time in order to show how these networks behavior develop and to determine the laws governing the evolution of physical systems. ex: Barabási and Albert for scale free networks [28].
- *Static models* show how networks are structured and how some properties of complex networks are present. The Watts and Strogatz model it is an example that explains the appearance of high clustering coefficient and low average path length in networks according to time [26].

There are several aspects in terms of the structure of a network that can be useful for predicting the overall behavior of a complex network, in terms of clusters how are interconnected, how to communicate with each other, how identify influential nodes in complex networks, how network structure affect the dynamics of social systems.

#### Social influence

The social influence is the most important topic in the field of complex system especially social network. We cannot talk about these type of networks without talking about spreading idea and information and the impact of this information on our society. Interactions between actors of social networks are the means by which information spreads. The maximizing of social influence is one of the issues concerning information propagation. It is essential to find a group of the most influential individuals in a social network so that they can extend their influence to the largest scale (influencers). In other words, the activation of these nodes can cause the propagation of information in the whole network. The problem of maximizing social influence has been an important research topic for many years because it has a considerable impact on the society. Some parties are interested by the progress in this area in order to optimize the spreading of information and new ideas through social network. Viral marketing is one of its application. The principle is that to promote a new service for all potential customers. The brand can target a limited group of clients who will subsequently tell their friends and acquaintances about the service. Other application of spreading information is the political company via social networks [29].

### Literature review on complex networks

In past decades, CN have gotten much consideration from researchers and nowadays, they have become a subject key in many areas of science. Studies on CN show that the modeling of these systems the complexity reduces to a level that we can manage them in a practical way [30]. The graphical properties produced by this modeling are similar to the real system [30]. There are various examples of complex networks in our daily life. Despite the fact that various measures have been suggested by researchers about complex networks, there are three basic metrics that can describe the characteristics of complex networks. These metrics are average path length [4], clustering coefficient and degree distribution. Degree distribution is a probabilistic distribution of the degrees of each node of the network [31]. Clustering coefficient evaluates the level of local or global transitivity of a graph. In other words, we study the links at the level of the triads (relations between three nodes) and we check whether, when there is a link between the nodes ab and bc, there is also a link between the nodes a and c. The average path length is the average length of shortest path between any two vertices [32].

Formerly, researches on complex networks focus on the topological structure of the network and its characteristics as well as its dynamics. The objective of studying and analyzing complex networks is not only to understand different real systems but also to achieve an effective control of these networks. In fact, to predict and control such a complex system or network an understanding of the mathematical description of these systems is necessary [30]. According to the dynamic process of complex networks, networks can be divided into two classes: static and temporal. The study of complex networks began with this class where the presence of nodes and links is unrelated from any idea of time. The static network contains nodes and edges altered gradually over times or fixed permanently. It is widely studied and suitable for analytical traceability [30]. In the dynamic class, the concept of time is relevant and the existence of links and nodes is time-sensitive, they are not always granted to exist. This kind of network is more realistic. Links between nodes in these networks may appear or disappear over time, the scientific collaboration network as an example [30]. There is a lot of sub-classes under these two classes (static-temporal). Networks can be distinguished according to their distribution degree, the average distance and other metrics. Models are developed through the year from simple lattices until improved models. Lattices are the simple models of networks. They are suitable for solving analytic problems [30] such as Ising Model [33] and Voter Model [34]. They have a simplified structure but are unrealistic in comparison with real-world systems [30], this is why the historical evolution of the models knows more improvement by taking into account more real characteristics. Afterward, in 1959 Erdos and Rényi explored another basic network mockup is the random regular network [35]. Watts and Strogatz [26] proposed the small-world model. It is more realistic and social network-inspired. Barabasi and Albert [13] developed a preferential attachment model that might be used to reproduce the time growth features of many real networks. Nodes are added in this model at each step by creating links with the already existing nodes with a proportional probability of their degrees at that moment. A model close to the BA (Barabasi and Albert) network was proposed by Bianconi and Barabasi [14] (Fitness Model). This model relies in addition to degree, on the fitness of each node for realizing new links. A new idea in BA models is introduced by Almeida et al. [13]. This idea is homophily, and models are christened homophilic model. Homophilic models rely on degree, fitness and also similarity between nodes for example similarity of jobs or similarity of interests, etc. Catanzaro et al. provide an algorithm for creating uncorrelated random networks (URN), despite the fact that this model is uncommon in real networks. URN is created in order to reach a theoretical solution for the behavior of dynamical systems. Waxman [36] suggested a generalization model of the Erdos–Renyi graph in 1988 (spatial Waxman Model). The challenge of building longer connections between nodes is fully considered in this model. Rozenfeld et al. [11] proposed the scale free on lattice. When creating new links, this model considers the Euclidean distance among nodes. Perra et al. [37] propose the activity driven model as an example of temporal social network. Actor activity drives relationships in this model. Afterward, the Adaptive networks model is appeared to give the same importance between the topology and the dynamical process [38]. Metapopulation model [39] also is presented as network constituted by collection of networks describing interconnected populations. Two level characterizing this model, the first is interpopulation that contain set of individuals and each individual constitutes the intrapopulation level. Multilayer model presents two layers (horizontal and vertical) which contain a two-way dynamic process within the layer and between layers [40]. Covid 19 is a good example to clarify this model as one of the infectious diseases which is contagious from bat animals to human. In this case, we can model human and their dynamic process as first layer and the same for bats animals as a second layer. There are human-to-human interactions, as there are human-to-animal relationships. Table 1 summarizes all of these network models. For each network model, we highlight its advantages as well as its limits.

**Table 1:** Network models and their characteristics

Authors	Networks models	Advantages	Limits
Jozef Sumec	Regular lattices	Simple models of networks. Suitable for solving analytic problems.	Unrealistic compared with real networks.
Erdos and Renyi [35]	Random regular network	Simple prototype of network, homogeneous.	Too restrictive.
Watts and Strogatz [26]	Small world networks	Realistic roused from social networks.	With a power-law basis, it is unable to construct heterogeneous degree distribution.
Barbasi and Albert [28]	Barbasi Albert model	Appropriate for generating the time growth characteristic among several real—world networks. Model of emergence graph.	The dynamic process is treated as static in this network. Fitness of nodes is not considered for making new links.
Bianconi and Barbasi [41]	Fitness model	Similar to BA model. Consider degree and fitness of nodes for making new connections.	Does not predict the impact of homophily.
Almeida et al. [42]	Homophilic model	Consider similitude of nodes. Model of emergence of small-world features and power-law degree distribution.	Produces undirected networks, It faces some difficulties in extending this model to directed networks.
Catanzaro et al. [43]	Uncorrelated random networks	It is important for checking theoretical solutions of the interactions of dynamical systems.	Unusual in real networks.
Waxman [36]	Spatial Waxman model	generalization of the Erdos–Renyi graph Consider geographical properties.	Weak in the prediction of most real systems.
Rozenfeld et al. [11]	Scale free on lattice	When creating new links, keep the Euclidean distance between nodes in consideration.	The entire length of the system's links can be kept to a minimum.
Perra et al. [37]	Activity driven model	Actor action drives relationships. Example of temporal social network.	Do not consider other features of actor activity like different weights associated with each connection.
Gross et al. [44]	Adaptive networks	Useful to model many real systems. With adaptive way, topologies change with changes of node’s states.	There is yet no clear theoretical explanation for large-scale adaptive network limitations.
Colizza and Vespignani [39]	Metapopulation model	A network of networks that describes a connected population. Widely used because of the mobility of node.	In spatial epidemiology, it is difficult to represent the essential aspects of spatial transmission of infectious diseases [45].
Mucha et al. [40]	Multilayer networks	The dynamic process has the potential to propagate inside and between layers.	The spectral characteristics of the graph can be used to identify distinct multiplexity regimes and coupling between layers [46].

In the last few years, there has been a growing interest in community structure and influential nodes in the field of complex network analysis. A large number of articles were published, including a different approach to the problem of community detection as in [47–53]. These referred approaches are classified as approach-based static non-overlapping communities, approach-based static overlapping communities, approach-based hierarchical communities and approach-based dynamic communities [54]. Researches are also interested in identifying influential nodes. Many approaches are proposed in this context as explained in the following section.

### Influential nodes finding approaches

In network science, each node plays a specific role. Nodes do not have the same importance, and some nodes are more important in the network than others remaining nodes due to their important capability of spreading in the whole network. These nodes are known as influential nodes. The identification of significant nodes is necessary in network attacks, network of terrorists, and disease spreading studies. Reason for what, approaches for finding important nodes in complex networks have attracted much interest. Several methods are proposed to identify these nodes: Degree centrality (DC) [55], betweenness centrality (BC),closeness centrality (CC) [56], page rank (PR) [57], Leader rank (LR) [58], H-index [59], Hyperlink-Induced Topic Search (HITS) [60], weighted formal concept analysis (WFCA) [61], weighted TOPSIS (W-TOPSIS) [62], Analytic hierarchy process AHP [63], Least-squares support vector machine LS-SVM [64]…. These proposed approaches are divided into four categories: structured approaches, vector-based approaches, MCDM-based approaches and machine learning-based approaches.

#### Structured approaches

In structured approaches, there are several types: local, semi-local, global and hybrid approaches. These techniques can also be classified into two classes: one is based on each node’s neighborhood (including degree centrality, K-shell, and H-index techniques), whereas the other is based on node pathways (such as closeness centrality and betweenness centrality). For local approaches, they determine the impact of nodes based on local data which means they depend on nodes and their neighbors to indicate their influence (impact). For example, H-index and degree centrality (DC), these approaches’ advantages are their simplicity and minimal computational complexity. However, the overall system structure is neglected and important nodes are found mostly in big components of multi-component [65], which diminishes the adequacy of these methods in extensive scale networks [66]. In global approaches, the importance of nodes is described by the entire structure of the network, e.g., closeness centrality (CC), betweenness centrality (BC), Coreness centrality (Cnc) [56], Kshell decomposition [59]. Centralities like closeness and betweenness are based on paths between nodes. These two measures are not as impactful in large-scale networks as a result of their great complexity of information, Kshell decomposition (Ks) indicates a global location features of network nodes but is not ideal for tree networks. Semi-local approaches use information on neighbors' neighbors (second-order neighbors) not withstanding information on neighbors to determine the spread capacity of a node. Example of these approaches: Weight Degree Centrality (*W*_DC_) [67] and Extended Weight Degree Centrality (EW_DC_) [10], in *W*_DC_ and EW_DC_ the computation of the diagrams assortativity is vital which can prompt more prominent time intricacy in vast scale graphs. Hybrid approaches use global information in conjunction with local information to specify these influential nodes and to determine the extended ability of these nodes, these techniques are based on the Ks index these methods, which are based entirely on the ks index include mixed degree decomposition [68], neighborhood coreness [69], k-shell iteration factor [66] and mixed core, degree and entropy [70].

#### Eigenvector-based approaches

Eigenvector-based approaches take into account the quantity of neighbors and their influences, such as: eigenvector centrality [71], Pagerank (PR) [57], LeaderRank (LR) [58], HITS (Hyperlink-Induced Topic Search) [60]. Eigenvector centrality can be productively determined utilizing a power iteration approach, yet it might end up caught in a zero status, on account of the presence of many nodes without in-degree [61]. PageRank is a variant of the eigenvector centrality. This famous algorithm is used in Google search engine. Firstly, acquainted with measure the ubiquity of a website page. It expects that the significance of a page is dictated by the amount and nature of the pages connected to it. It has been used in several areas and works well in networks without scale. However, it is sensitive to disturbances of random networks and presents thematic drifts in special network structures [61]. The HITS algorithm considers every node in the system by including two jobs: the authority and the hub similarly HITS introduces a wonder of topical drift. LeaderRank works well in complex directed networks but seems to be inapplicable on non-directed complex networks.

#### MCDM-based approaches

Recently, multi-criteria analysis methods (MCDMs) or multiple attribute decision making methods (MADMs) have been used to classify nodes according to their importance, like TOPSIS [14] W-TOPSIS [62] and AHP [63]. Various measurements of centrality have been utilized as multiple attributes of complex networks. However, each attribute assumes an imperative job in TOPSIS, which is not sensible, to cure this issue W-TOPSIS not just considers diverse centrality measures as multiple network attributes, However, it also suggests a new technique for calculating the weight of each attribute. AHP is also applied to detect important nodes and uses the model susceptible-Infected SI to obtain the weights. Yang also mixes entropy with TOPSIS to generate EW—TOPSIS [72]. In this combination, TOPSIS is based on centrality measures as multi-criteria and the entropy is used to calculate the weight of each factor.

#### Machine learning-based approaches

Recently, there has been a significant focus on machine learning-based approaches. Least Square Support Vector Machine (LS-SVM) was used by Wen et al. to identify the mapping rules among basic indicators and AHP performance evaluation [64]. LS-SVM furnishes good supervision for identifying important nodes in large-scale networks. Zhao et al. proposed a model to identify vital nodes based on seven algorithms of machine learning (Naïve Bayes, Decision Tree, Random Forest, Support Vector Machine SVM, K-Nearest Neighbor KNN, Logistic Regression, and Multi-layer Perceptron MLP). This model relies on graph model and rate of infection in the ranking of nodes. Approaches based on machine learning rely a lot on feature engineering, and the selection of these features can influence the performance of these approaches. To handle this task, Zhao et al. [73] introduced a deep learning model called infGCN. It is based on graph convolutional networks. InfGCN treat in the same time the features of node and the link between them.

### Classification summary

In this section, we present different well-known approaches that are used to identify influential nodes and we perform a comparison between them based on some factors. The selected approaches do not present an exhaustive list of research on influential finding nodes approaches. In this comparison, we will focus on the type, the nature and the direction of network used in the approach. The network’s type indicates whether the network is weighted or unweighted. The network's nature indicates whether it is static or dynamic. The network direction indicates whether or not the network is directed. Network size provides the size of the used network. The implementation datasets present datasets used in the implementation of the approach. Table 2 presents the abbreviation and its description for the used complex networks datasets for each technique implementation.

**Table 2:** Operational network datasets implemented in the main comparison's referred research

Networks dataset	Common abbreviation	Description
LFR benchmark	LFR	Lancichinetti–Fortunato–Radicchi benchmark (An artificial network produced by the LFR algorithm that resembles a real-world network).
Zebra	ZBR	Animal network that contains interactions between 28 Grévy's zebras (*Equus grevyi*) in Kenya. Zebras are represented by nodes, and an edge between two zebras indicates that there was interaction between them during the study.
Zachary karate club	ZKC	Human Social network of university of karate club that gathers students of the club of karate by Wayne Zachary in 1977. Each node represents a member of the club, and each edge represents a tie between two members of the club.
Contiguous	CTG	The contiguous zone, the marin boundary between 12NM (Nautical miles) and 24NM.
Dolphins	DLP	A social network of bottlenose dolphins. The nodes are the bottlenose dolphins (genus *Tursiops*) of a bottlenose dolphin community living off Doubtful Sound, a fjord in New Zealand (spelled *fiord* in New Zealand). An edge indicates a frequent association. The dolphins were observed between 1994 and 2001.
Copperfield	CPF	Network of common word (adjacencies between noun and adjectives) for the novel David Copperfield by Charles Dickens. Nodes represent the most commonly occurring adjectives and nouns in the book. Edges connect any pair of words that occur in adjacent position in the text of the book.
Co authorship in network science	NTS	Co-authorship of scientists in network theory and experiments.
*Caenorhabditis elegans*	ELG	Neural network of neurons and synapses in *C. elegans*, a type of worm. It consists of around 1000 cells including 302 neurons.
Euroroad	ERD	A international E-road network located mostly in Europe. Network includes cities, and an edge connecting two cities indicates that they are linked. It contains 1174 cities.
Chicago	CCG	Contains a comprehensive list of all current City of Chicago workers with details.
Hamsterster	HMS	Network is of the friendships and family links between users of the website http://www.hamsterster.com. It is an independent site created in 2003 or 2004. Hamsterster appears to have been shut down as of October 2014.
US power grid	UG	Undirected infrastructure network provides data concerning the Western States of the USA of America's power grid. An edge represents a power supply line. A node is either a generator, a transformator or a substation.
Pretty good privacy	PGP	An online contact network or an interaction network of users of the pretty good privacy (PGP) algorithm. The network contains only the giant connected component of the network.
Astro physics	ASP	Collaboration or cooperation network based on the e-print arXiv and includes scientific partnerships between authors of articles submitted to the Astro Physics field. If an author *i* co-authored a paper with author *j*, the graph contains a undirected edge from *i* to *j*. The data covers papers in the period from January 1993 to April 2003 (124 months). It begins within a few months of the inception of the arXiv, and thus represents essentially the complete history of its ASTRO-PH section.
Enron email network	ENR	The Enron email dataset comprises about 500,000 emails sent by Enron Corporation employees. This data was originally made public, and posted to the web, by the Federal Energy Regulatory Commission during its investigation. Nodes of the network are email addresses and if an address *i* sent at least one email to address *j*, the graph contains an undirected edge from *i* to *j.*
Jazz musicians	JZ	Collaboration network between Jazz artists. Each node represents a Jazz artist, and each edge indicates that two artists have collaborated in a band. Two levels of collaborations are studied. First, the collaboration network between individuals, where two musicians are connected if they have played in the same band and second, the collaboration between bands, where two bands are connected if they have a musician in common.
Email network of URV	URV	The email communication network of the University Rovira I Virgili in Tarragona, Catalonia, Spain. Nodes are users and each edge represents that at least one email was sent. The direction of emails and the number of emails between two persons are not stored.
BLOGS	BG	Communication network between users of MSN’s (windows live) blog. It’s composed of 3982 nodes and 6803 edges.
COND-MAT (condense matter physics)	CoundMath	Collaboration network based on the e-print arXiv and includes research partnerships between authors who have submitted articles to the Condense Matter category. If an author *i* co-authored a paper with author *j*, the graph contains a undirected edge from *i* to *j*. If the paper is co-authored by *k* authors this generates a completely connected (sub) graph on *k* nodes. The data covers papers in the period from January 1993 to April 2003 (124 months). It begins within a few months of the inception of the arXiv, and thus represents essentially the complete history of its COND-MAT section.
Live journal	LJ	Free online blogging community with almost 10 million members where individuals express their friendship toward others. LiveJournal allows members to maintain journals, individual and group blogs, and it allows people to declare which other members are their friends they belong.
Contact network of inpatients	CNI	Presents link between two inpatients if they have both been admitted to the same hospital.
Internet Movie database actors in adult films	IMDB	Network of connections between actors who have co-starred in films, whose genre has been labeled by the Internet Movie Database as ‘adult’. The dataset is a bipartite graph in which each node either corresponds to an actor or to a movie. Edges go from a movie to each actor in the movie. It also provides metadata for the nodes like movie/actor name, year of the movie, and genre of the movie.
Email contact network	EM	The network of email contacts is formed on email messages sent and received at University College London's Computer Sciences Department.
The Internet at the router level (RL)	RL	The nodes of the RL Internet network are the Internet routers. Two routers are connected if there exists a physical connection between them.
The Internet at the autonomous system level (AS)	AS	The nodes are autonomous systems that are linked if there is a real connection beyond them. graph of routers comprising the Internet can be organized into sub-graphs called Autonomous Systems (AS). Each AS exchanges traffic flows with some neighbors (peers). We can construct a communication network of who-talks-to- whom from the BGP (Border Gateway Protocol) logs. The data was collected from University of Oregon Route Views Project—Online data and reports. The dataset contains 733 daily instances which span an interval of 785 days from November 8 1997 to January 2 2000. In contrast to citation networks, where nodes and edges only get added (not deleted) over time, the AS dataset also exhibits both the addition and deletion of the nodes and edges over time.
Product space of economic goods	PS	Is a network that formalizes the idea of relatedness between products traded in the global economy. Proximity network between products according to Ref.
Word	WAN	Represents an adjacency relation in English text.
*E. coliproteins*	ECP	Presents the problem of identifying *E.coli* proteins based on amino acid sequences in cell localization regions. It contains 336 *E.coli* proteins split into 8 different classes.
Tandem affinity purification	TAP	Yeast protein–protein binding network generated by tandem affinity purification experiments.
Yeast 2 hybrid	Y2H	Yeast protein–protein binding network generated using yeast two hybridization. It is originally created by Fields and Song. Is a genetic system wherein the interaction between two proteins of interest is detected via the reconstitution of a transcription factor and the subsequent activation of reporter genes under the control of this transcription factor.
Power	PWR	Connections between power stations.
Internet (router level)	Int	Symmetrized snapshot of the Internet ‘s structure at the level of autonomous systems, the network size is 22963.
Facebook	FB	This *dataset* consists of friends lists from *Facebook*. Nodes represents actors or friends and edge represent the relationship between them.
Twitter	TW	Microblogging social network operated by the company Twitter Inc. It allows a user to send free text messages, called tweets, over the internet, by instant messaging or by SMS.
The John Padgett—Florentine Families Dataset	JPFF	Multiplex network with 2 edge types representing marriage alliances and business relationships between Florentine families during the Italian Renaissance. Data hosted by Manlio De Domenico. Marriage and commercial links between Renaissance Florentine families are represented in this dataset.
Delicious.com	DLC	Feature network. This dataset includes labeled web pages obtained from the website delicious.com. Left nodes represent tags, right nodes represent URLs and an edge shows that a URL was tagged with a tag.
UsairPort	UP	Network of direct flights linking US airports in 2010. Each edge represents a connection from one airport to another, and the weight of an edge shows the number of flights on that connection in the given direction, in 2010.
AirLines	AL	Flight arrival and departure data for all commercial flights from 1987 to 2008.
American College Football Network	ACF	Interaction network that represents Football games between Division IA institutions during the regular season in the Fall 2000.
Yeast	YST	Metabolic network. The dataset consists of a protein–protein interaction network. Research showed that proteins with a high degree were more important for the survival of the yeast than others. A node represents a protein and an edge represents a metabolic interaction between two proteins. The network contains loops.
Router	RTR	Routing network composed of 5022 nodes and 12 516 connections.
Human protein	HP	A network of protein–protein interactions that includes physical contacts between proteins that have been experimentally demonstrated in humans, such as metabolic enzyme-coupled interactions and signaling interactions. Nodes represent human proteins and edges represent physical interaction between proteins in a human cell.
General relativity and quantum cosmology collaboration network	CA-GrQc	The collaboration network derives from the e-print arXiv and contains scientific partnerships between authors on articles submitted to the category of General Relativity and Quantum Cosmology. If an author *i* co-authored a paper with author *j*, the graph contains a undirected edge from *i* to *j*. The data covers papers in the period from January 1993 to April 2003 (124 months). It begins within a few months of the inception of the arXiv, and thus represents essentially the complete history of its GR-QC section.
High energy physics theory collaboration network	Ca-HepTh	collaboration network is from the e-print arXiv and covers scientific collaborations between authors papers submitted to High Energy Physics—Theory category. If an author *i* co-authored a paper with author *j*, the graph contains a undirected edge from *i* to *j*. If the paper is co-authored by *k* authors this generates a completely connected (sub)graph on *k* nodes. The data covers papers in the period from January 1993 to April 2003 (124 months). It begins within a few months of the inception of the arXiv, and thus represents essentially the complete history of its HEP-TH section.
Groad	GRD	Highway network of 1168 nodes.

For the benchmarking approaches used in the detection of influential nodes, a list of real and artificial networks is presented above in Table 2. This step of benchmarking is important to see how the approach or the algorithm is efficient, and also, it can give us the ability to compare results of different approaches on the same dataset.

We give, in the following comparison table, examples of employed implementation datasets (refer to Table 2) in each specified reference, as well as other features as follows:

The following comparison offers an overview of the most widely used techniques in this problematic of influential node’s detection. All of these techniques show their effectiveness throw various experimentation and produce results differentiated by their calculation, limitations, complexity, time of execution, nature and size of network.

In this table, there are some approaches that are in the same spirit for example PageRank and HITS. Both of them utilize the connection structure of the Web graph to determine the pertinence of the pages. HITS works on small subgraph representing the connection between hub and authority websites from the webgraph which explains their complexity that is inferior of $O \left( {\log N} \right).$ The limitations of PageRank are that does not account for time; also, it is unable to handle advanced search queries. It is unable to analyze a text in its entirety while searching for keywords. Instead, Google interprets these requests and filters search results using natural language processing NLP. From these experiments on the datasets mentioned above, there are some methods that have low time complexity, for example, the k-shell algorithm, HKS, MDD, KS-IF, and Cnc, their time complexity is $O\left( n \right)$ where n is the number of edges in the network. The k-shell decomposition approach was initially developed for unweighted undirected networks, but it has lately been expanded to other kinds of networks. The k-shell approach was expanded by Garas et al. [74] to recognize core-periphery structure in weighted networks. In K-shell decomposition, the K-shell value is not an appropriate metric for measuring influence. The k-shell index's monotonicity is lower than other centrality indices. MDD is proposed to remedy the problem of the k-shell method where the exhausted degree, as well as the residual degree, are taken into account. AHP, TOPSIS, and W-TOPSIS also have the same philosophy to aggregate centralities to evaluate the influence of nodes. They consider local information and global structure to identify influential nodes. TOPSIS is implemented under four real directed and undirected networks, and it demonstrates their practicability. AHP is implemented under four real undirected networks, and the SI model is used to confirm the accuracy of ranking nodes using AHP. This method outperforms W-TOPSIS. W-TOPSIS is extended to dynamic networks in other work by Pingle Yang et al. [75]. LS-SVM is implemented on an artificial network using two network models: WS-small world network and power-law BA scale-free network, real networks also are used in an implementation like the US aviation network, dolphin social network, American college football, netscience, and email network. LS-SVM reduced the computation-intensive evaluation of node importance to a basic calculation of the nodes' basic indicators. infGCN proves its accuracy on five different real networks (different types and sizes). Experimental results on these networks indicate that InfGCN can strongly increase prediction accuracy.

The topology characteristics of the networks have an effect on the index accuracy. The performance of the same index varies among networks. In some situations, it can be challenging to select the indices that will best identify the influential nodes. Therefore, finding influential nodes is still a current unresolved problem.

## Conclusion

In this paper, a short review of complex networks is presented. Some taxonomy around complex networks is summarized, like the structure of networks, measurements of the network, and social influence within networks. A literature review is provided including the evolution of networks and models through the years, from simple lattices to more complex models. The pros and cons of each model are highlighted with some references for those who want to go further with this issue. In addition, we provide a detailed comparison review between approaches used to identify influential nodes as mentioned above in Table 3. Throw this comparison, this paper clarifies some strengths of each approach in order to help beginner researchers in this field to identify the relevant directives for their future contributions to this problem of influential node identification. This given work of literature review does not cover all available works related to the identification of influential nodes. Although dynamic networks rely on variations in characteristics and the emergence of properties of networks over time, the majority of approaches are applied to static networks rather than dynamic ones. It really requires working on dynamic networks again. From future perspectives, we can adapt existing approaches of identifying influential nodes to dynamic networks. Additionally, we can combine existing methods with the aim of taking advantage of both methods and achieving a balance between them as we can combine machine learning and deep learning algorithms with other methods.

**Table 3:** Influential nodes finding approaches comparison

References	Approach	Network type	Network nature	Network direction	Network size	Implementation datasets
[76]	HKS	Unweighted/weighted	Static	Undirected	All	LFR, ZBR, ZKC, CTG, DLP, CPF,NTS, ELG, ERD, CCG, HMS, UG, PGP, ASP, ENR
[69]	Coreness centrality (Cnc)	Unweighted	Dynamic	Undirected	All	ZKC, DLP, JZ,ELG,NTS, URV, BG, UG, BA, LFR PG, ASP, CA-CondMat, ENR, EM
[59]	Kshell decomposition	Weighted/Unweighted	Dynamic	Directed/undirected	Medium and large	LJ, EM, CNI, IMDB, CondMat RL, AS, PS
[68]	Mixed degree decomposition (MDD)	Unweighted	Static	Undirected	All	DLP, JZ, NTS, EM, Ca-HepTh, PGP, ASP CondMat, WAN, ECP, ELG, TAP, Y2H, PWR, Int
[66]	k-shell iteration factor (KS-IF)	Unweighted	Dynamic	Undirected	All	LFR,ZKC,DLP, JZ, NTS, EM, BG, PGP, ENR,FB, TW
[77]	Eigenvector centrality	Unweighted	Static	Directed	Small	JPEF
[57]	PageRank	Unweighted/weighted	Dynamic	Directed	Large	Google search Engine
[58]	LeaderRank	Unweighted	Static	Directed	Large	DLC
[60]	HITS	Weighted/unweighted	Dynamic	Directed	Small	Clever search engine
[78]	TOPSIS	Unweighted	Static	Undirected, directed	Medium and large	UP, AL, EM, ACF
[17]	W-TOPSIS	Unweighted	Static	Undirected	Large	YST, BG, RTR, PGP
[63]	AHP	Unweighted	Static	Undirected	Medium and large	EM, GRD, YST, UP
[64]	LS-SVM	Unweighted/Weighted	static	Undirected/directed	All	WS small-world network, power-law, BA scale-free network, UP, DLP,ACF, NTS, EM
[73]	infGCN	Unweighted	Static	Undirected	Large	HMS, HP, CA-GrQc, CA-HepTh, CondMat

## Data Availability

The datasets used and/or analyzed during the current study are available from the corresponding author on reasonable request.

## Abbreviations

CN: Complex networks
MCDM: Multi-criteria decision making
WS: Watts and Strogatz
BA: Barabasi and Albert
DC: Degree centrality
BC: Betweenness centrality
CC: Closeness centrality
PR: PageRank
LR: LeaderRank
HITS: Hyperlink-induced topic search
WFCA: Weighted formal concept analysis
W-TOPSIS: Weighted TOPSIS
AHP: Analytic hierarchy process
LS-SVM: Least-squares support vector machine
Cnc: Coreness centrality
Ks: Kshell decomposition
W_DC_: Weight degree centrality
EW_DC_: Extended weight degree centrality
MADMs: Multiple attribute decision making methods
EW–TOPSIS: Entropy weighted TOPSIS
SVM: Support vector machine
KNN: K-nearest neighbor
MLP: Multi-layer perceptron
MDD: Mixed degree decomposition
KS-IF: K-shell iteration factor
NLP: Natural language processing

## References

[1] Arqub OA, Abo-Hammour Z (2014). Numerical solution of systems of second-order boundary value problems using continuous genetic algorithm. Inf Sci.

[2] Abo-Hammour Z, Alsmadi O, Momani S, Abu Arqub O (2013). A genetic algorithm approach for prediction of linear dynamical systems. Math Probl Eng.

[3] Abo-Hammour Z, Abu Arqub O, Momani S, Shawagfeh N (2014). Optimization solution of Troesch’s and Bratu’s problems of ordinary type using novel continuous genetic algorithm. Discrete Dyn Nat Soc.

[4] Ahuja M, Sharma K (2014). Complex networks: a review. Int J Comput Appl.

[5] Abu Arqub O, Abo-Hammour Z, Momani S, Shawagfeh N (2012). Solving singular two-point boundary value problems using continuous genetic algorithm. Abstr Appl Anal.

[6] Mahdy AMS, Higazy M, Mohamed M (2021). Optimal and memristor-based control of a nonlinear fractional tumor-immune model. Comput Mater Contin.

[7] Mahdy AMS (2022). A numerical method for solving the nonlinear equations of Emden-Fowler models. J Ocean Eng Sci.

[8] Ismail GM, Mahdy AMS, Amer YA, Youssef ESM (2022). Computational simulations for solving nonlinear composite oscillation fractional. J Ocean Eng Sci.

[9] Mahdy AMS, Lotfy K, El-Bary AA (2022). Use of optimal control in studying the dynamical behaviors of fractional financial awareness models. Soft Comput Fusion Found Methodol Appl.

[10] Mahdy AMS, Lotfy K, El-Bary A, Sarhan HH (2021). Effect of rotation and magnetic field on a numerical-refined heat conduction in a semiconductor medium during photo-excitation processes. Eur Phys J Plus.

[11] Rozenfeld AF, Cohen R, Ben-Avraham D, Havlin S (2002). Scale-free networks on lattices. Phys Rev Lett.

[12] Mahdy AMS, Lotfy K, El-Bary A, Tayel IM (2021). Variable thermal conductivity and hyperbolic two-temperature theory during magneto-photothermal theory of semiconductor induced by laser pulses. Eur Phys J Plus.

[13] Milgram S (1967) The small-world problem

[14] Mahdy AMS, Babatin MM, Khader MM (2022). Numerical treatment for processing the effect of convective thermal condition and Joule heating on Casson fluid flow past a stretching sheet. Int J Mod Phys C.

[15] Mahdy AMS, Lotfy K, El-Bary A (2022). Thermo-optical-mechanical excited waves of functionally graded semiconductor material with hyperbolic two-temperature. Eur Phys J Plus.

[16] Mahdy AM, Amer YAE, Mohamed MS, Sobhy E (2020). General fractional financial models of awareness with Caputo-Fabrizio derivative. Adv Mech Eng.

[17] Mahdy AMS, Mohamed MS, Lotfy K, Alhazmi M, El-Bary AA, Raddadi MH (2021). Numerical solution and dynamical behaviors for solving fractional nonlinear Rubella ailment disease model. Results Phys.

[18] Réseau sans échelle, *hmn.wiki*. https://hmn.wiki/fr/Scale-free_networks (consulté le 9 mars 2022).

[19] Mahdy A, Higazy M (2019). Numerical different methods for solving the nonlinear biochemical reaction model. Int J Appl Comput Math.

[20] Mahdy AMS, Mohamed DS (2022). Approximate solution of Cauchy integral equations by using Lucas polynomials. Comput Appl Math.

[21] Khader MM, Swetlam NH, Mahdy AMS (2014). The Chebyshev collection method for solving fractional order Klein-Gordon equation. Wseas Trans Math.

[22] Higazy M, El-Mesady A, Mahdy AMS, Ullah S, Al-Ghamdi A (2021). Numerical, approximate solutions, and optimal control on the deathly lassa hemorrhagic fever disease in pregnant women. J Funct Spaces.

[23] da F. Costa L, Rodrigues FA, Travieso G, Villas Boas R (2007). Characterization of complex networks: a survey of measurements. Adv Phys.

[24] Dijkstra EW, Beauguitte L, Maisonobe M, Dijkstra EW (1959) A note on two problems in connexion with graphs. Numerische Mathematik 1, 269271 Version bilingue commentée, février 2021. Consulté le: 9 mars 2022. [En ligne]. Disponible sur: https://hal.archives-ouvertes.fr/hal-03171590

[25] Ebel H, Davidsen J, Bornholdt S (2003) Dynamics of social networks, *ArXivcond-Mat0301260*, Consulté le: 9 mars 2022. [En ligne]. Disponible sur: http://arxiv.org/abs/cond-mat/0301260

[26] Watts DJ, Strogatz SH (1998). Collective dynamics of “small-world” networks. Nature.

[27] Liu Z, Jiang C, Wang J, Yu H (2015). The node importance in actual complex networks based on a multi-attribute ranking method. Knowl-Based Syst.

[28] Barabasi A-L, Albert R (1999). Emergence of scaling in random networks. Science.

[29] Daliri Khomami MM, Rezvanian A, Bagherpour N, Meybodi MR (2018). Minimum positive influence dominating set and its application in influence maximization: a learning automata approach. Appl Intell.

[30] Ang´ elica Sousa da Mata, Complex Networks: a Mini-review.

[31] Lu S (2018). Complex network description of the ionosphere. Nonlinear Process Geophys.

[32] Yen CC, Yeh MY, Chen MS (2013) An efficient approach to updating closeness centrality and average path length in dynamic networks. In: 2013 IEEE 13th international conference on data mining, Dallas, TX, USA, 2013, pp 867–876. 10.1109/ICDM.2013.135

[33] Brush SG (1967). History of the Lenz-Ising model. Rev Mod Phys.

[34] Frachebourg L, Krapivsky L (1996). Exact results for kinetics of catalytic reactions. Phys Rev E.

[35] Erdos, Renyi A (1958) On random graphs I., pp 290–297

[36] Waxman BM (1988). Routing of multipoint connections. IEEE J Sel Areas Commun.

[37] Perra N, Gonçalves B, Pastor-Satorras R, Vespignani A (2012). Activity driven modeling of time varying networks. Sci Rep.

[38] Guo Q, Lei Y, Jiang X, Ma Y, Huo G, Zheng Z (2016). Epidemic spreading with activity-driven awareness diffusion on multiplex network. Chaos Interdiscip J Nonlinear Sci.

[39] Colizza V, Vespignani A (2007). Invasion threshold in heterogeneous metapopulation networks. Phys Rev Lett.

[40] Mucha J, Richardson T, Macon K, Porter MA, Onnela J (2010). Community structure in time-dependent, multiscale, and multiplex networks. Science.

[41] Bianconi G, Barabási A-L (2001). Competition and multiscaling in evolving networks. EPL Europhys Lett.

[42] de Almeida ML, Mendes GA, Madras Viswanathan G, da Silva LR (2013). Scale-free homophilic network. Eur Phys J B.

[43] Catanzaro M, Boguñá M, Pastor-Satorras R (2005). Generation of uncorrelated random scale-free networks. Phys Rev E.

[44] Adaptive Networks PDF - brodistaniperro7. https://sites.google.com/a/hz.books-now.com/en180/9783642012846-49vovecGEerin51 (consulté le 24 octobre 2021).

[45] Wang L, Li X (2014). Spatial epidemiology of networked metapopulation: an overview. Chin Sci Bull.

[46] Aleta A, Moreno Y (2019). Multilayer networks in a nutshell. Annu Rev Condens Matter Phys.

[47] Lancichinetti A, Fortunato S, Radicchi F (2008). Benchmark graphs for testing community detection algorithms. Phys Rev E.

[48] Fortunato S (2010). Community detection in graphs. Phys Rep.

[49] Radicchi F, Castellano C, Cecconi F, Loreto V, Parisi D (2004). Defining and identifying communities in networks. Proc Natl Acad Sci.

[50] Newman MEJ, Girvan M (2004). Finding and evaluating community structure in networks. Phys Rev E.

[51] Xie J, Szymanski BK (2012) Towards linear time overlapping community detection in social networks, *ArXiv12022465 Phys.*, févr. 2012, Consulté le: 5 octobre 2021. [En ligne]. Disponible sur: http://arxiv.org/abs/1202.2465

[52] Bai L, Liang J, Du H, Guo Y (2018). A novel community detection algorithm based on simplification of complex networks. Knowl-Based Syst.

[53] A community discovering method based on event network for topic detection.

[54] El-Moussaoui M, Agouti T, Tikniouine A, Adnani ME (2019). A comprehensive literature review on community detection: approaches and applications. Procedia Comput Sci.

[55] Freeman LC (1978). Centrality in social networks conceptual clarification. Soc Netw.

[56] Borgatti S, Everett MG (2000). Models of core/periphery structures. Soc Netw.

[57] Brin S, Page L (2012). Reprint of: The anatomy of a large-scale hypertextual web search engine. Comput Netw.

[58] Lü L, Zhang Y-C, Yeung CH, Zhou T (2011). Leaders in social networks, the delicious case. PLoS ONE.

[59] Kitsak M (2010). Identification of influential spreaders in complex networks. Nat Phys.

[60] Prajapati MR (2012). A survey paper on hyperlink-induced topic search (HITS) algorithms for web mining. Int J Eng.

[61] Sun Z, Wang B, Sheng J, Hu Y, Wang Y, Shao J (2017). Identifying influential nodes in complex networks based on weighted formal concept analysis. IEEE Access.

[62] Hu J, Du Y, Mo H, Wei D, Deng Y (2016). A modified weighted TOPSIS to identify influential nodes in complex networks. Phys Stat Mech Appl.

[63] Bian T, Hu J, Deng Y (2017). Identifying influential nodes in complex networks based on AHP. Phys Stat Mech Appl.

[64] Wen X, Tu C, Wu M, Jiang X (2018). Fast ranking nodes importance in complex networks based on LS-SVM method. Phys Stat Mech Appl.

[65] Sheikhahmadi A, Nematbakhsh MA, Shokrollahi A (2015). Improving detection of influential nodes in complex networks. Phys Stat Mech Appl.

[66] Wang Z, Zhao Y, Xi J, Du C (2016). Fast ranking influential nodes in complex networks using a k-shell iteration factor. Phys Stat Mech Appl.

[67] Kanwar K, Kaushal S, Kumar H (2019). A hybrid node ranking technique for finding influential nodes in complex social networks. Libr Hi Tech.

[68] Zeng A, Zhang C-J (2013). Ranking spreaders by decomposing complex networks. Phys Lett A.

[69] Bae J, Kim S (2014). Identifying and ranking influential spreaders in complex networks by neighborhood coreness. Phys Stat Mech Appl.

[70] Sheikhahmadi A, Nematbakhsh MA (2017). Identification of multi-spreader users in social networks for viral marketing. J Inf Sci.

[71] Estrada E, Rodríguez-Velázquez JA (2005). Subgraph centrality in complex networks. Phys Rev E.

[72] Node importance ranking in complex networks based on multicriteria decision making.

[73] Gouheng Zhao et al, InfGCN: Identifying influential nodes in complex networks with graph convolutional networks

[74] Garas A, Schweitzer F, Havlin S (2012). A k-shell decomposition method for weighted networks. New J Phys.

[75] Yang P, Liu X, Xu G (2018). A dynamic weighted TOPSIS method for identifying influential nodes in complex networks. Mod Phys Lett B.

[76] Zareie A, Sheikhahmadi A (2018). A hierarchical approach for influential node ranking in complex social networks. Expert Syst Appl.

[77] Lloyd B (2001). Eigenvector-like measures of centrality for asymmetric relations. Soc Netw.

[78] Du Y, Gao C, Hu Y, Mahadevan S, Deng Y (2014). A new method of identifying influential nodes in complex networks based on TOPSIS. Phys Stat Mech Appl.

